# Effects of Active-Center Reduction of Plant-Type Ferredoxin on Its Structure and Dynamics: Computational Analysis Using Molecular Dynamics Simulations

**DOI:** 10.3390/ijms232415913

**Published:** 2022-12-14

**Authors:** Tomoki Nakayoshi, Yusuke Ohnishi, Hideaki Tanaka, Genji Kurisu, Hiroko X. Kondo, Yu Takano

**Affiliations:** 1 Graduate School of Information Sciences, Hiroshima City University, 3-4-1 Ozukahigashi, Hiroshima 731-3194, Japan; 2 Institute for Protein Research, Osaka University, 3-2 Yamadaoka, Suita 565-0871, Japan; 3 Faculty of Engineering, Kitami Institute of Technology, 165 Koen-cho, Kitami 090-8507, Japan

**Keywords:** ferredoxin, metalloprotein, [2Fe-2S] cluster, force field, molecular dynamics simulation

## Abstract

“Plant-type” ferredoxins (Fds) in the thylakoid membranes of plants, algae, and cyanobacteria possess a single [2Fe-2S] cluster in active sites and mediate light-induced electron transfer from Photosystem I reaction centers to various Fd-dependent enzymes. Structural knowledge of plant-type Fds is relatively limited to static structures, and the detailed behavior of oxidized and reduced Fds has not been fully elucidated. It is important that the investigations of the effects of active-center reduction on the structures and dynamics for elucidating electron-transfer mechanisms. In this study, model systems of oxidized and reduced Fds were constructed from the high-resolution crystal structure of *Chlamydomonas reinhardtii* Fd1, and three 200 ns molecular dynamics simulations were performed for each system. The force field parameters of the oxidized and reduced active centers were independently obtained using quantum chemical calculations. There were no substantial differences in the global conformations of the oxidized and reduced forms. In contrast, active-center reduction affected the hydrogen-bond network and compactness of the surrounding residues, leading to the increased flexibility of the side chain of Phe61, which is essential for the interaction between Fd and the target protein. These computational results will provide insight into the electron-transfer mechanisms in the Fds.

## 1. Introduction

**Figure 1:**
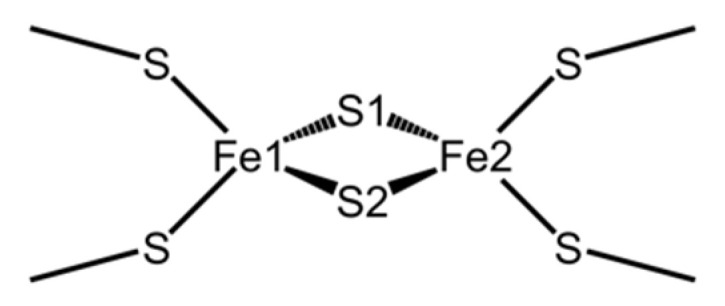
Chemical structure of the [2Fe-2S] cluster.

**Figure 2:**
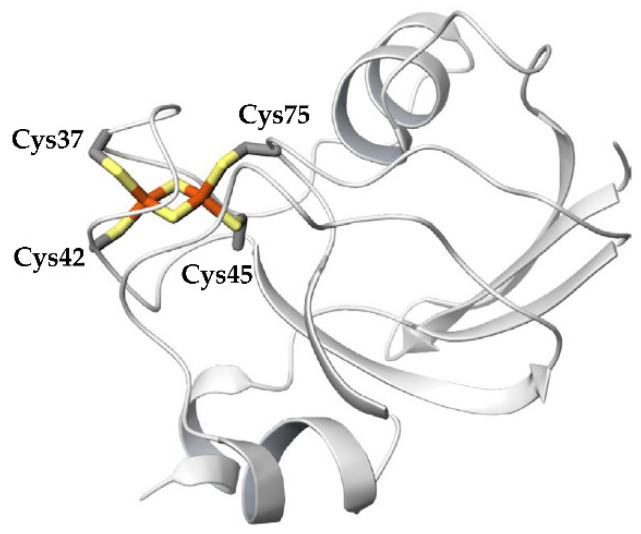
Overall structure of plant-type Fd. [2Fe-2S] cluster and ligated Cys residues are presented as licorice.

Ferredoxins (Fds) are electron-transfer proteins that contain iron-sulfur clusters as active centers and are present in various prokaryotic and eukaryotic organisms. Fds are roughly classified into four types based on the number of iron and sulfur atoms in the cluster: [1Fe-0S], [2Fe-2S], [3Fe-4S], and [4Fe-4S] [1]. Fds in plants, algae, and cyanobacteria are called “plant types” and contain a single [2Fe-2S] cluster ligated by four highly conserved cysteine (Cys) residues. As shown in Figure 1, each iron atom in the [2Fe-2S] cluster is coordinated by two side-chain sulfhydryl sulfur atoms of Cys residues and inorganic sulfur atoms, and the four sulfurs are placed tetrahedrally around each iron atom. Plant-type Fds are globular proteins consisting of approximately 100 residues (Figure 2) and are rich in acidic amino acid residues. Most plant-type Fds have extremely low redox potentials of the [2Fe-2S]^2+/+^ transition, between −390 and −425 mV [2]. To date, the amino acid sequences of various plant-type Fds have been determined, and they have high sequence similarities.

The electronic states of [2Fe-2S] clusters were characterized using various spectroscopic studies [3]. Under physiological conditions, [2Fe-2S] clusters mainly adopt two types of electronic states, that is, oxidized and reduced states [3]. In the oxidized state, both Fe atoms are in a sextet high-spin Fe^3+^ state (*S* = 5/2), giving rise to *S* = 0 ground states. In the reduced state, one Fe atom is in a quintet high-spin Fe^2+^ state (*S* = 2), and the other is in a sextet high-spin Fe^3+^ state, giving rise to *S* = 1/2 ground states. Dugad et al. revealed that the iron atom located closer to the molecular surface (labeled as Fe1 in Figure 1) of the two iron atoms in plant and algae derived Fds was reduced [4].

Light reactions occur in the thylakoid membranes during photosynthesis in plants, algae, and cyanobacteria [5]. The electron transport chains in the thylakoid membranes are composed of three membrane protein complexes: Photosystem II (PS II), cytochrome *b_6_f*, and Photosystem I (PS I). The plastoquinone and plastocyanine act as single-electron carriers. When PS II is photoactivated, two water molecules supply four electrons to PS II, producing oxygen molecule as a by-product. Electrons are transferred from PS II to PS I via plastoquinone, cytochrome *b_6_f*, and plastocyanine. Plant-type Fds mediate light-induced electron transfer from the PS I active center to various Fd-dependent enzymes such as Fd-NADP^+^ reductase (FNR), Fd-thioredoxin reductase, and nitrile reductase [6]. The affinity between Fds and the target proteins depend on the redox state. The oxidized Fd is preferentially bound to PS I, however the reduced Fd rapidly dissociates from PS I [7]. In addition, in *Equisetum arvense*, the affinities of oxidized Fd and FNR are approximately 18-fold lower than those of reduced Fd and FNR [8]. Such redox-dependent affinities are believed to contribute to smooth electron transfer.

Structural biological studies on plant-type Fds have been conducted for a long time. Fukuyama et al. reported the three-dimensional (3D) structure of plant-type Fd for the first time [9]. They determined the X-Ray crystal structure of Fd from *Spirulina platensis* at a resolution of 2.5 Å, registered in the Protein Data Bank (PDB) (PDB ID: 4FXC, updated to 1FXC via 3FXC) [9,10,11]. Since then, many high-resolution crystal structures of oxidized Fds, such as those of polypodiophyta *Equisetum arvense* Fd2 at 1.20 Å (PDB ID: 1WRI) [8], thermophilic cyanobacteria *Mastigocladus laminosus* Fd at 1.25 Å (PDB ID: 1RFK) [12], and cyanobacteria *Anabaena* PCC7119 at 1.30 Å (PDB ID: 1QT9) [13] have been determined. The first reported crystal structure of reduced Fd was from *Anabaena* PCC 7119 at a resolution of 1.17 Å (PDB ID: 1CZP) [13]. It has been previously suggested that strong X-Ray irradiation can cause partial reductions of redox centers in metalloproteins [14]. Therefore, the crystal structures of the oxidized plant-type Fds reported to date are suspected to be mixtures of the oxidized and reduced states. Mixed redox states can hinder the perception of the 3D structures of Fds. Recently, Ohnishi, et al., determined the X-Ray crystal structure of the *Chlamydomonas reinhardtii* Fd1 (*Cr*Fd1) at a resolution of 0.90 Å (PDB ID: 6LK1) [15], making it the plant-type Fds with the highest resolution currently registered in PDB. Moreover, the crystallographic and spectroscopic analyses revealed that the active centers underwent X-Ray-dose-dependent reductions. However, the partial active-center reductions induced by X-Ray irradiation did not cause substantial conformational changes in the Fds [15]. The structural knowledge of plant-type Fds is relatively limited to static ones, and the detailed behavior of oxidized and reduced Fds is yet to be fully elucidated. Since the redox states of Fds can significantly change the affinities for target proteins, the accurate structures of the oxidized and reduced forms in solution may play important roles in understanding the interactions between Fds and target proteins. Computational methods are useful for understanding events that are difficult to analyze experimentally [16]. In this study, molecular dynamics (MD) simulations were performed on model systems constructed from the crystal structures of *Cr*Fd1 to elucidate the structural and dynamic properties of fully oxidized and reduced Fds in solution.

## 2. Results and Discussion

**Figure 3:**
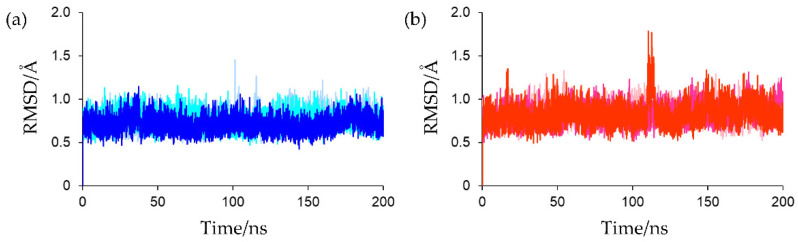
(**a**) RMSD plots of protein backbone for oxidized Fd. RMSD plots of the first, second, and third simulations were presented in light blue, cyan, and blue, respectively. (**b**) RMSD plots of protein backbone for reduced Fd. RMSD plots of the first, second, and third simulations were presented in light pink, magenta, and red, respectively.

The MD software AMBER does not provide force field parameters for [2Fe-2S] clusters. Therefore, the force field parameters for the oxidized and reduced [2Fe-2S] clusters were calculated independently using quantum chemical calculations. The model systems were constructed from the crystal structure of *Cr*Fd1 (PDB ID: 6LK1), and three independent 200 ns MD simulations were performed for the oxidized and reduced forms. To confirm the convergence of the MD simulations, the root mean square deviation (RMSD) values for the protein backbones were calculated. The crystal structure was used as the reference structure for RMSD calculations. As shown in Figure 3, all simulations converged in the early stages and continued up to 200 ns without significant fluctuations, suggesting that all the simulations proceeded in a stable manner and that the calculated structures of both the oxidized and reduced forms did not differ from the crystal structures. In the third simulation for the reduced form, a local increase in the RMSD value was observed around 110–115 ns. This was attributed to a large movement of the *C*-terminal region, and the global protein conformation remained almost unchanged. The average RMSD values for the final 80 ns MD trajectories of the first, second, and third simulations for oxidized Fds were 0.787 ± 0.093, 0.758 ± 0.088, and 0.707 ± 0.084 Å, respectively, and those for reduced Fds were 0.835 ± 0.103, 0.858 ± 0.103, and 0.840 ± 0.121 Å, respectively. To assess the validities of the parameters determined by quantum chemical calculations, the structures extracted from the final 80 ns MD trajectories were compared with the experimental structures. The calculated structures of the oxidized forms were compared with the crystal structures of *Cr*Fd1 (PDB ID: 6KUM) [15] and *Anabaena* PCC7119 Fd (PDB ID: 1QT9) [13]. The 3D structure of PDB entry 6KUM was determined by low-dose X-ray irradiation and with minimal X-ray damage. The calculated structures of the reduced forms were compared with those of *Cr*Fd1 (PDB ID: 6KV0) [15] and *Anabaena* PCC7119 Fd (PDB ID: 1CZP) [13]. In PDB entries 6KV0 and 1CZP, the Fd structures were partially reduced by high-dose X-ray irradiation and the addition of reducing agents, respectively. As shown in Tables S1 and S2, the average bond lengths and angles in the structures obtained by simulations were similar to those of the experimental structures, indicating the validity of the parameters determined by quantum chemical calculations.

**Figure 4:**
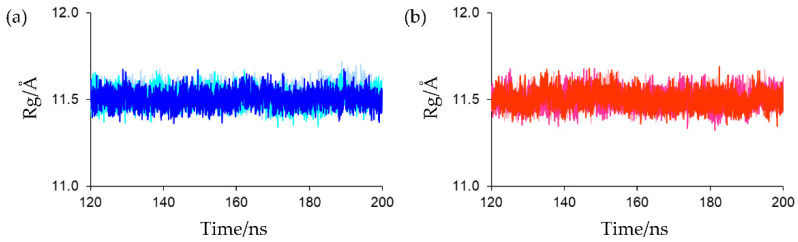
(**a**) Rg plots of protein backbone for oxidized Fd. Rg plots of the first, second, and third simulations were presented in light blue, cyan, and blue, respectively. (**b**) Rg plots of protein backbone for reduced Fd. Rg plots of the first, second, and third simulations were presented in light pink, magenta, and red, respectively.

Overall rigidity and compactness were evaluated using the radius of gyration (Rg). The Rg value for the protein backbone in the crystal structure (PDB entry: 6LK1) was 11.345 Å. The Rg plots for the final 80 ns MD trajectories are shown in Figure 4. As shown in this figure, no substantial differences were observed in the Rgs at equilibrium states for all simulations (the average Rg values for the final 80 ns MD trajectories of the first, second, and third simulations for oxidized Fds were 11.528 ± 0.047, 11.508 ± 0.047, and 11.507 ± 0.045 Å, respectively, and those for reduced Fds were 11.505 ± 0.049, 11.503 ± 0.048, and 11.503 ± 0.049 Å, respectively). This indicates that both the oxidized and reduced forms in solution were as compact as the crystal structure and the rigidities and compactness of the oxidized and reduced forms were.

**Figure 5:**
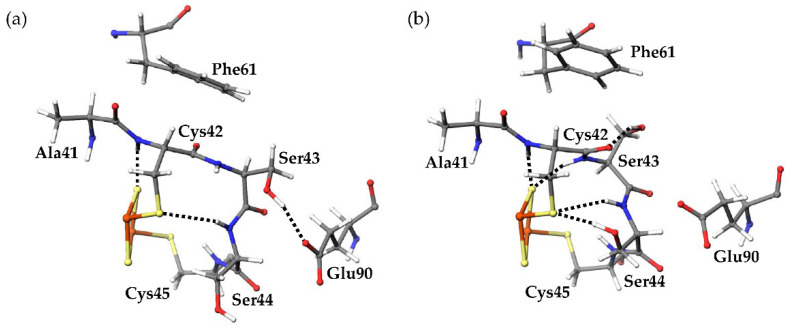
Structural differences around the active site between (**a**) oxidized and (**b**) reduced forms. The hydrogen bonds are presented as dotted lines. The hydrogens, carbons, nitrogens, oxygens, sulfurs, and irons are displayed in white, gray, red, yellow, and orange, respectively, in a ball-and-stick model.

**Figure 6:**
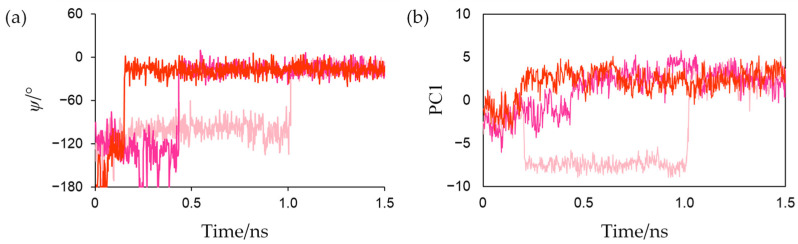
(**a**) Time courses of dihedral angle *ψ* characterizing the main-chain conformation of Cys42 in reduced forms and (**b**) time courses of PC1. Plots of the first, second, and third simulations are presented in light pink, magenta, and red, respectively.

Around the active center of plant-type Fds, it was observed that the inorganic sulfurs in the [2Fe-2S] cluster (denoted as S1 and S2 in Figure 1) and the side-chain sulfhydryl groups of ligated Cys residues formed multiple [NH…S] and [OH…S] hydrogen bonds with the main-chain amide and side-chain hydroxy groups of surrounding residues. These hydrogen bonds are considered to be important for keeping the redox potential extremely low [17]. Here, the amino acid residues (Tyr35-Cys45 and Thr74-Cys75) which formed [NH…S] and [OH…S] hydrogen bonds with the [2Fe-2S] cluster are defined as “residues around the active site.” The most remarkable structural difference around the active site was the flip of the peptide bond linking Cys42 and Ser43 (Figure 5). In this study, the initial structures of the simulations for both oxidized and reduced forms were constructed from the crystal structures of oxidized *Cr*Fd1, with the main-chain carbonyl group of Cys42 pointing toward the [2Fe-2S] cluster (“CO-in” conformation). The main-chain carbonyl group formed the “CO-in” conformation in the oxidized form equilibrated in solution, while it was oriented on the “opposite” side in the equilibrated reduced form (“CO-out” conformation). These results indicate that flips of peptide bonds linking Cys42 and Ser43 occurred during MD simulations of the reduced form. Figure 6a shows the changes in the dihedral angle *ψ* (N-Cα-C-N) that characterizes the main-chain conformation of Cys42 in reduced forms. The *ψ* values rapidly increased at approximately 0.15, 0.45, and 1.01 ns in the first, second, and third simulations, respectively, and did not significantly fluctuate afterwards. This indicates that the peptide bond was irreversibly flipped in the early stage and that the flips were thermally promoted. Corresponding peptide-bond flips have also been experimentally observed in Fds from *Anabaena* PCC7119 [15]. This computational result suggests that MD simulations can reproduce the conformational changes caused by active-center reduction.

To evaluate the conformational changes near the active center of Fd that occur in conjunction with the peptide-bond flip during active-center reduction, MD trajectories from the start of the production MD simulation to 1.5 ns of the three simulations for the reduced form were merged, and principal component analysis (PCA) was performed in the Cartesian coordinates of all the heavy atoms of residues around the active site and Phe61. The contribution of the first eigenvector (PC1) was 37.9%, and the main-chain atoms of Cys42 and Ser43 and the main- and side-chain atoms of Phe61 predominantly moved along PC1 as shown in Figure S1. Changes in the projections of each trajectory onto the PC1 along time are shown in Figure 6b. This indicates that the peptide-bond flip and the orientation change of the Phe61 occurred in tandem.

**Figure 7:**
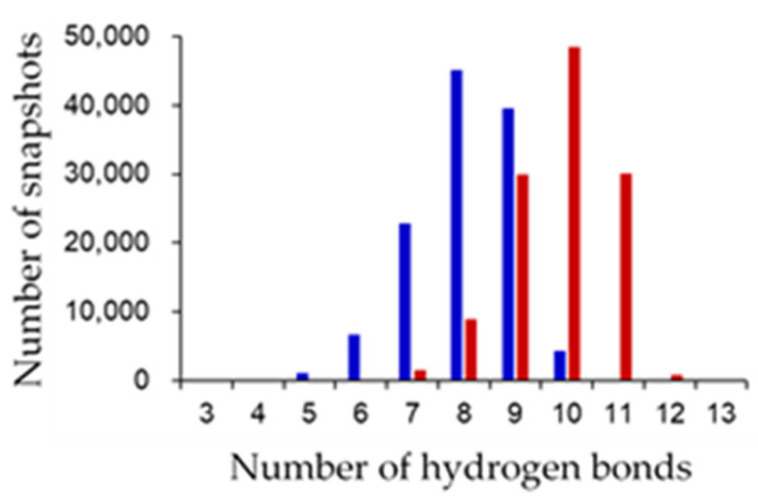
Distribution of the number of [NH…S] and [OH…S] hydrogen bonds. The number of hydrogen bonds in the oxidized and reduced forms are represented in blue and red, respectively.

Several differences were observed in the hydrogen-bond network around the active sites between the oxidized and reduced forms. The final 80 ns MD trajectories of three independent simulations were merged for the oxidized or reduced form and used for hydrogen-bond analysis. The “highly frequent” [NH…S] and [OH…S] hydrogen bonds (occurrence frequencies >40%) observed in the oxidized and reduced forms are shown in Table 1. Figure 7 shows the distribution of the numbers of [NH…S] and [OH…S] hydrogen bonds. The average numbers of [NH…S] and [OH…S] hydrogen bonds were 8.069 ± 0.997 and 9.821 ± 0.967 in the oxidized and reduced forms, respectively. The [NH…S] and [OH…S] hydrogen bonds in the reduced form were enhanced compared to those in the oxidized forms, with a few exceptions. The flip of the peptide bond linking Cys42 and Ser43 in the reduced form resulted in significant changes in the hydrogen-bond network. In the reduced form, an additional hydrogen bond between the main-chain amide group of Ser43 and S2 was observed with an occurrence frequency of 73.49%. This is because the main-chain amide NH group of Ser43 did not point toward the [2Fe-2S] cluster in the oxidized form, whereas it pointed toward the reduced form. In addition, in the oxidized form, the side-chain hydroxy group of Ser43 formed a hydrogen bond with the side-chain carboxy group of Glu90 at an occurrence frequency of 53.40%. On the other hand, a hydrogen bond was formed in the reduced form with the main-chain carbonyl group of Cys42 at an occurrence frequency of 63.31% and with the side-chain carboxy group of Glu90 at less than 1% occurrence frequency. The peptide-bond flip brought the main-chain carbonyl group of Cys42 closer to the side-chain hydroxy group of Ser43, inducing changes in these hydrogen-bond pairs. While the side-chain hydroxy group of Ser44 did not form highly frequent hydrogen bonds with specific residues, a hydrogen bond was formed with the side-chain sulfhydryl sulfur of Cys42 at an occurrence frequency of 76.40%. The change in the orientation of the side chain of Ser44 is considered to occur because the active center in the reduced form was more negatively charged than that in the oxidized form, resulting in an enhanced Coulomb interaction. In contrast, active-center reduction weakened the hydrogen bonds between the main-chain amide group of Cys37 and the side-chain sulfhydryl group of Cys42 and between the main-chain amide group of Gly40 and the side-chain sulfhydryl group of Cys75. While the main-chain amide group of Cys37 formed only highly frequent hydrogen bonds with the side-chain sulfhydryl group of Cys42 in the oxidized form (at a frequency of 76.96%), this hydrogen bond was somewhat destabilized in the reduced form, and instead a highly frequent hydrogen bond formed with S1. To assess the direct effects of the peptide-bond flip on the hydrogen-bond network, MD trajectories from the start of the production MD simulation to 1.5 ns of the three simulations for the reduced form were merged, and the occurrence frequencies of the hydrogen-bond formation before and after peptide-bond flip were compared. The side-chain hydroxy group of Ser43 formed hydrogen bonds with the side-chain carboxyl group of Glu90 at 89.2% before the flip, while at 1.58% after the flip. In addition, the occurrence frequency of hydrogen bond between the main-chain amide group of Ser43 and S2 was 1.00% before the flip, however 75.0% after the flip. These indicate that the flip of peptide bond linking Cys42 and Ser43 clearly affect the hydrogen-bond network near the active site.

**Figure 8:**
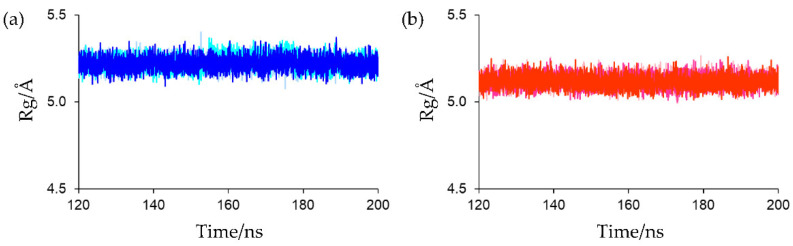
(**a**) Rg plots of protein backbone around the active site for oxidized Fd. Rg plots of the first, second, and third simulations were presented in light blue, cyan, and blue, respectively. (**b**) Rg plots of protein backbone around the active site for reduced Fd. Rg plots of the first, second, and third simulations were presented in light pink, magenta, and red, respectively.

The Rg of the main-chain heavy atoms of these residues was calculated to assess the compactness and rigidity of the residues around the active sites. The Rg plots for the final 80 ns MD trajectories are shown in Figure 8. The average Rg values of the final 80 ns MD trajectories of the first, second, and third simulations for oxidized Fds were 5.220 ± 0.036, 5.228 ± 0.038, and 5.220 ± 0.036 Å, respectively, and those for reduced Fds were 5.120 ± 0.035, 5.119 ± 0.035, and 5.117 ± 0.035 Å, respectively. This indicates that the active-site peripheries of the oxidized and reduced forms have similar rigidities and that the residues around the active site in the reduced form were slightly more compact than those in the oxidized form. The higher compactness of the reduced form may be because of the enhanced [NH…S] hydrogen bonds. However, despite differences in compactness around the active sites, there was little differences in overall protein compactness between the oxidized and reduced forms, as shown in Figure 4. This may be due to the fact that the active site is located on the protein surface, as shown in Figure 2, and that the size of the active sites is much smaller than that of the overall protein.

**Figure 9:**
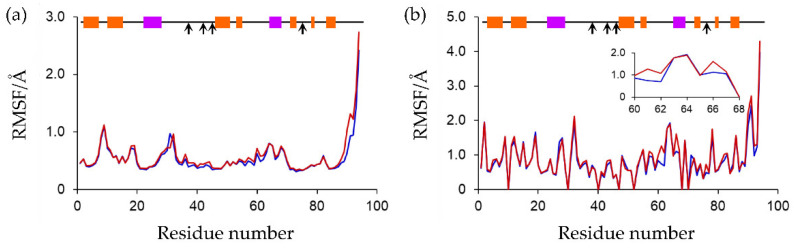
RMSF plot of (**a**) main-chain heavy atoms and (**b**) side-chain heavy atoms. RMSF values of side chains of Gly residues were taken as 0. RMSF values of side-chain heavy atoms of residues 60–68th are presented in the inner panel. Plots of the oxidized and reduced Fds are presented in blue and red, respectively. In the horizontal bars at the top of the plots, α-helices and β-sheets are indicated in purple and orange. The positions of the ligated Cys residues are indicated by arrows.

Root mean square fluctuation (RMSF) values were calculated to evaluate the flexibility of each residue. The final 80 ns MD trajectories of the three simulations were merged for the oxidized or reduced form and used for the RMSF analysis. The average mass-weighted RMSF values for the main-chain and side-chain heavy atoms were independently calculated. The RMSF plots are shown in Figure 9. In both the oxidized and reduced forms, the main-chain RMSF values of almost all residues were within 1 Å, indicating that the protein backbones were very rigid, as suggested by Rg calculations. In addition, there were no substantial differences in the protein-backbone flexibilities between the oxidized and reduced forms, except for the *C*-termini. While the side-chain flexibilities were similar for most residues, the side chains of Phe61, Leu62, and Gln66 of the reduced form were more flexible than those of the oxidized form (inner panel of Figure 9b). As shown in Figure 5, Phe61 faces Ala41-Ser43 around the active site. It was conceivable that the active-center reduction caused the structural changes around the active sites, which includes peptide-bond flips, recombination of the hydrogen-bond networks. In addition, it increased the compactness because of the enhanced [NH…S] hydrogen bonds, directly affecting the side-chain flexibility of Phe61. In the crystal structure of the complex of Fd and FNR from *Anabaena* PCC7119 (PDB ID: 1EWY), the corresponding Phe residue was present at the Fd/FNR interface [18]. Moreover, the hydrophobic interactions involving this Phe residue play an important role in forming the Fd-FNR complex [19,20,21]. Therefore, the change in orientation of the side chain of Phe61 in *Cr*Fd1 can affect its interaction with FNR. Previously, it was experimentally confirmed that the affinity between Fd1 and FNR from *Equisetum arvense* decreased approximately 18-fold after electron transfer from Fd1 to FNR [7], and this affinity change is considered to contribute to smooth electron transfer. Taking this into consideration, the aforementioned structural changes and changes in the side-chain orientation and flexibility of Phe61 induced by active-center reduction can affect the affinities of the target proteins and might explain parts of the electron-transfer mechanism.

## 3. Materials and Methods

The high-resolution crystal structure at 0.90 Å of *Cr*Fd1 has been registered as entry 6LK1 in PDB [15]. The force field parameters of the active centers of the oxidized and reduced states were determined using quantum chemical calculations. These were independently determined to be consistent with the AMBER force fields using Models 1 and 2, which were constructed from PDB entry 6LK1. The structures of Models 1 and 2 are shown in Figure S2. Model 1 contained a [2Fe-2S] cluster and four ligated Cys residues (Cys37, Cys42, Cys45, and Cys75); the *N*- and *C*-termini were replaced with hydrogen atoms. Model 2 contained a [2Fe-2S] cluster and residues around the active site. In Model 2, the side chains of the four ligated Cys residues were explicitly considered, and those of other amino acid residues were replaced by hydrogen atoms. In addition, the *N*- and *C*-termini of the peptide chains were capped with acetyl and methylamino groups, respectively. Model 1 was used to calculate the force constants of bond stretching and angle bending, and Model 2 was used to calculate the partial atomic charges. For Model 1, geometry optimizations were performed without any constraints using density functional theory methods with LC-ωPBE exchange-correlational functional [22]. In addition, vibrational frequency calculations were performed for the optimized geometry. In the geometry optimizations and vibrational frequency calculations, different basis sets were applied depending on the elements; 6-31 + G(df,p) for Fe, 6-31 + G(d) for S, and 6-31G(d) for H, C, N, and O. Tables S3 and S4 present the coordinates of the optimized geometries of Model 1 in the oxidized and reduced states, respectively. Based on the Seminario method [23], the force constants of bond stretching, and angle bending were determined from Hessian matrices. The force constants of the torsion motion were set to zero, as in several previous studies [24,25,26,27]. The electrostatic potentials for Model 2 were calculated using the Hartree-Fock method with 6-31G(d) basis sets. The atomic charges at the peptide bonds in Model 2 were manually set to common values of the AMBER ff14SB force field [28], and those of other atoms were determined by fitting the electrostatic potentials based on the RESP methods [29]. The parameters used for calculations of the oxidized and reduced forms are summarized in Tables S5 and S6, respectively. Models 1 and 2 were constructed and force constants were calculated using the MCPB.py program of AmberTools [30]. All quantum chemical calculations were performed using Gaussian16 software [31].

The initial model system for MD simulations was constructed based on PDB entry 6LK1. The atomic coordinates of chain A and its corresponding [2Fe-2S] cluster were extracted from entry 6LK1 and was used as simulation templates. That is, the crystal water and other solute molecules were not included in the initial model systems. The protonation states of the side chains of the amino acid residues at pH 7.0 were determined based on p*K*_a_ values calculated using the H++ web server [32]. In p*K*_a_ calculations, the internal and external dielectric constants of proteins were set to 10 and 80, respectively (which is the default setting in the H++ web server). The *N*-terminal Ala residue added for crystallization was first removed. The system was neutralized by adding Na^+^ ions and was solvated using TIP3P water models [33] in a truncated octahedral box with a minimum distance of 10 Å between the solute and box edges. The initial structures of the oxidized anda reduced forms contained 3343 and 3342 water molecules, respectively. First, water molecules and Na^+^ ions were optimized, whereas the heavy atoms of the protein and [2Fe-2S] cluster were restrained with a force constant of 500 kcal mol^−1^ Å^−2^. All atoms were optimized without any constraints. After these optimizations, the system was heated from 0 to 300 K for 200 ps (30 K/20 ps) with weak position restraints applied to the heavy atoms of the protein and [2Fe-2S] cluster with force constants of 10 kcal mol^−1^ Å^−2^. Subsequently, a total of 500-ps MD simulations were performed with the protein-backbone restraints to relax the side-chain conformations. In this process, the peptide-backbone restraints were gradually reduced after every 100 ps, and the force constants at 0–100, 100–200, 200–300, 300–400, and 400–500 ps were 10, 5.0, 1.0, 0.50, and 0.10 kcal mol^−1^ Å^−2^, respectively. Finally, to equilibrate the entire system, 200 ns production MD simulations were performed. Three simulations with different initial velocities were conducted for each system. During the relaxation and production MD simulations, the system pressure was maintained at 1 bar using a Berendsen barostat [34] and the system temperature was regulated at 300 K using a Langevin thermostat [35]. The AMBER ff14SB force field [28] was used for the amino acid residues, and the parameters of the [2Fe-2S] cluster and the four ligated Cys residues were taken from the parameters obtained by quantum chemical calculations, as mentioned above. The periodic boundary conditions were applied to the systems, and long-range electrostatic interactions were efficiently computed using the particle mesh Ewald method [36]. The cutoff distance for the non-bonded-term calculations was set to 10 Å. The stretching motions of all covalent bonds linking the hydrogen and heavy atoms were constrained using the SHAKE algorithm [37]. Geometry optimization and MD simulations were performed using AMBER20 [38].

Snapshots were sampled every 2 fs, and the final 80 ns MD trajectories (containing 40,000 snapshots for each trajectory) were used for analysis. The main-chain heavy atoms of all snapshots were superimposed on those of the crystal structure, and the RMSD and Rg values were calculated. In addition, the occurrence frequencies of hydrogen bonds in each snapshot were calculated. For this analysis, the occurrence of hydrogen bonds was verified if the following two conditions were satisfied: The distance between the heavy atoms of the donor and acceptor was within 4.0 Å, and the angle between the donor, proton, and acceptor was greater than 140°. For RMSF analysis, the coordinates of the main-chain heavy atoms of the snapshots sampled from each MD trajectory were averaged, and the RMSF values were calculated after the main-chain heavy atoms of each snapshot were superimposed on those of the average structure. The RMSDs, Rgs, hydrogen bonds, and RMSFs were analyzed using the cpptraj module of AmberTools [39].

## 4. Conclusions

In this study, 200 ns MD simulations were performed for the oxidized and reduced forms to investigate the effects of active-site reduction on the structure and dynamics of *Cr*Fd1 using model systems constructed from the crystal structures of *Cr*Fd1. All simulations reached equilibrium in the early stages, and the structures of the oxidized and reduced forms in solution were not substantially different from the crystal structures. The calculations of Rg and RMSF values for main-chain heavy atoms of all amino acid residues indicated that the main chains of both the oxidized and reduced forms were rigid, and their rigidities were very similar. However, there were significant differences in the structures around the active centers between the oxidized and reduced forms. As observed in the crystal structures of *Anabaena* PCC7119 Fds [12], the thermal-promoted flips of peptide bonds linking Cys42 and Ser43 occurred in the reduced forms in the early stages of MD simulations, which showed that the standard NPT MD simulations could accurately reproduce the conformational changes induced by Fd reduction. The peptide-bond flips and the increase in negative charges at the active centers result in local hydrogen-bond recombination and the enhancement of [NH…S] hydrogen bonds. The enhanced [NH…S] hydrogen bonds made the vicinities of the active sites more compact and contributed to the increased flexibility of the side chain of Phe61. Phe61 is considered to be important for forming the Fd-FNR complex [18,19,20]. Since the 3D structures of *Chlamydomonas reinhardtii* FNR has not been experimentally determined, it was not possible to determine the specific effects of the orientation and flexibility of Phe61 in *Cr*Fd1 on the formation and dissociation of the Fd-FNR complexes. In *Anabaena* PCC7119, it has been experimentally suggested that the side-chain orientation of the Phe65 (equivalent to Phe61 in *Cr*Fd1) in conjunction with the flip of the peptide bond linking Cys46 and Ser47 (equivalent to Cys42 and Ser43 in *Cr*Fd1, respectively) plays an important role in the formation and dissociation of the Fd-FNR complex [40]. As shown in Figure 6b and Figure S1, PCA indicated that the active-site reduction altered the orientation of Phe61, which supports the experimental data and the differences in side-chain flexibility between the oxidized and reduced forms may explain the difference in affinity for FNR between oxidized and reduced states.

To date, structural biological studies of plant-type Fds have revealed many of their properties and functions. While many crystal structures of plant-type Fds have been determined, most of these are oxidized forms, and the structural information on the reduced Fds are limited. This study shows that the normal NPT MD simulations with appropriate force field parameters can be used to obtain the structures of reduced Fds from the experimental structures of oxidized Fds. Our computational results are expected to be useful for modeling the complexes of Fds and target proteins. In addition, owing to experimental difficulties, the positions of hydrogen atoms around the active sites have not been clarified.

In a recent theoretical study, Era et al., showed that the number of [NH…S] and [OH…S] hydrogen bonds affect the redox potentials of plant-type Fds [41], suggesting the importance of the position of a hydrogen atom. In addition, they showed that the active center of reduced form is relatively stabilized by hydrogen bonds and that the stronger charge polarization of main-chain amide NH group shifts the redox potential. Our computational results showed that the residues around active site in the reduced form are more compact than those in oxidized form, indicating an overall increase in the strength of the [NH…S] hydrogen bonds. The enhancements of [NH…S] hydrogen bonds can be primarily attributed to enhanced electrostatic interactions, which can contribute to the stabilization of the active center. The computational results in this study provide information about the enhancement or weakening of the [NH…S] and [OH…S] hydrogen bonds upon active-site reduction and proton positions of the side-chain hydroxy group of Ser44 and are expected to be useful for further investigation of the Fd properties. While MD simulations were performed for Fd alone in this study, it is essential to conduct molecular mechanical and quantum chemical studies on the complexes of Fds and the target proteins to elucidate the electron-transfer mechanisms in detail. We plan to conduct a computational study for these complexes in our future study.

## Figures and Tables

**Table 1:** Highly frequent [NH…S] and [OH…S] hydrogen bonds in oxidized and reduced forms.

Hydrogen Bonds	Occurrence Frequencies	
	Oxidized Form	Reduced Form
Cys37 S_γ_ ← Ala39 N	95.83%	97.16%
Cys37 S_γ_ ← Ala41 N	86.46%	90.98%
Cys42 S_γ_ ← Cys37 N	76.96%	46.68%
Cys42 S_γ_ ← Ser44 N	81.78%	97.91%
Cys42 S_γ_ ← Ser44 O_γ_	10.85%	76.40%
Cys45 S_γ_ ← Cys75 N	94.66%	91.45%
Cys75 S_γ_ ← Gly40 N	80.16%	66.62%
S1 ← Ser36 N	98.88%	99.39%
S1 ← Cys37 N	14.55%	46.31%
S1 ← Arg38 N	86.80%	94.89%
S2 ← Cys42 N	74.80%	94.71%
S2 ← Ser43 N	0%	73.49%

## Data Availability

The data presented in this study are available in the article and Supplementary Materials. The atomic coordinates of the crystal structure of *Cr*Fd1 were downloaded from PDB.

## Supplementary Materials

The following supporting information can be downloaded at: https://www.mdpi.com/article/10.3390/ijms232415913/s1, Table S1: Comparison of the calculated structures of oxidized forms obtained by MD simulations and the crystal structures; Table S2: Comparison of the calculated structures of reduced forms obtained by MD simulations and the crystal structures; Figure S1: The dominant motion along PC1 up to 1.5 ns after the start of the production MD simulation; Figure S2: Geometry of Models 1 and 2 constructed based on the crystal structure by MCPB.py; Table S3: Cartesian coordinates of optimized geometry of Model 1 with oxidized state; Table S4: Cartesian coordinates of optimized geometry of Model 1 with reduced state; Table S5: Input files for calculating oxidized *Cr*Fd1; Table S6: Input files for calculating reduced *Cr*Fd1.
