# Supplementary material for: Effects of Active-Center Reduction of Plant-Type Ferredoxin on Its Structure and Dynamics: Computational Analysis Using Molecular Dynamics Simulations
Source: Int J Mol Sci. 2022 Dec 14;23(24):15913. doi: 10.3390/ijms232415913 (PMC9782105; doi:10.3390/ijms232415913)
Supplement: Supplementary file 1 [file ijms-23-15913-s001.zip › ijms-2035668-supplementary.pdf]

Supplementary Materials

Effects of Active-Center Reduction of Plant-Type Ferredoxin on Its Structure and Dynamics: Computational Analysis Using Molecular Dynamics Simulations

Tomoki Nakayoshi [1,*], Yusuke Ohnishi [2], Hideaki Tanaka [2], Genji Kurisu [2], Hiroko X. Kondo [1,3], and Yu Takano [1,*]

[1] Graduate School of Information Sciences, Hiroshima City University, 3-4-1 Ozukahigashi, Asaminami-ku, Hiroshima, Hiroshima 731-3194, Japan

[2] Institute for Protein Research, Osaka University, 3-2 Yamadaoka, Suita, Osaka 565-0871, Japan
[3] Faculty of Engineering, Kitami Institute of Technology, 165 Koen-cho, Kitami, Hokkaido 090-8507, Japan

* Correspondence: nakayoshi@hiroshima-cu.ac.jp (T.N.); ytakano@hiroshima-cu.ac.jp (Y.T.); Tel.: +81-82-830-1736 (T.N.); +81-82-830-1852 (Y.T.)

Table S1. Comparison of the calculated structures of oxidized forms obtained by MD simulations and the crystal structures. Sγ1, Sγ2, Sγ3, and Sγ4 are represented the side-chain sulfhydryl sulfurs of Cys37, Cys42, Cys45, and Cys75 in *Cr*Fd1 (Cys41, Cys46, Cys49, Cys79 in *Anabaena* PCC7119 Fd), respectively.

Bond or angle	Calculated structure	6KUM	1QT9
Fe1–S1	2.174 ± 0.048	2.215	2.278
Fe1–S2	2.135 ± 0.048	2.172	2.236
Fe2–S1	2.198 ± 0.050	2.187	2.226
Fe2–S2	2.152 ± 0.050	2.145	2.192
Sγ1–Fe1	2.269 ± 0.059	2.345	2.338
Sγ2–Fe1	2.275 ± 0.061	2.298	2.278
Sγ3–Fe2	2.251 ± 0.058	2.329	2.288
Sγ4–Fe2	2.205 ± 0.058	2.303	2.290
Fe1–S1–Fe2	74.396 ± 1.912	75.542	75.063
S1–Fe2–S2	103.407 ± 2.169	104.289	105.604
Fe2–S2–Fe1	76.139 ± 1.990	77.307	76.574
S2–Fe1–S1	104.810 ± 2.178	102.435	102.452
Sγ1–Fe1–S1	116.185 ± 4.143	120.104	119.858
Sγ1–Fe1–S2	103.680 ± 3.929	102.484	102.156
Sγ2–Fe1–S1	104.033 ± 3.905	109.458	108.074
Sγ2–Fe1–S2	117.345 ± 4.536	117.413	119.133
Sγ1–Fe1–Sγ2	110.720 ± 4.222	105.471	105.917
Sγ3–Fe2–S1	114.982 ± 3.649	112.029	112.396
Sγ3–Fe2–S2	109.435 ± 4.200	111.497	111.213
Sγ4–Fe2–S1	112.659 ± 3.960	118.009	115.997
Sγ4–Fe2–S2	109.448 ± 3.778	104.276	104.842
Sγ3–Fe2–Sγ4	106.438 ± 4.447	106.494	106.574

Table S2. Comparison of the calculated structures of reduced forms obtained by MD simulations and the crystal structures. Sγ1, Sγ2, Sγ3, and Sγ4 are represented the side-chain sulfhydryl sulfurs of Cys37, Cys42, Cys45, and Cys75 in *Cr*Fd1 (Cys41, Cys46, Cys49, Cys79 in *Anabaena* PCC7119 Fd), respectively.

Bond or angle	Calculated structure	6KV0	1CZP
Fe1–S1	2.265 ± 0.062	2.239	2.285
Fe1–S2	2.212 ± 0.063	2.183	2.218
Fe2–S1	2.164 ± 0.049	2.164	2.198
Fe2–S2	2.155 ± 0.048	2.187	2.233
Sγ1–Fe1	2.204 ± 0.085	2.393	2.361
Sγ2–Fe1	2.281 ± 0.087	2.337	2.3
Sγ3–Fe2	2.301 ± 0.070	2.312	2.313
Sγ4–Fe2	2.220 ± 0.070	2.338	2.297
Fe1–S1–Fe2	73.903 ± 2.230	75.955	75.174
S1–Fe2–S2	106.013 ± 2.547	104.373	105.505
Fe2–S2–Fe1	75.157 ± 2.295	77.437	76.624
S2–Fe1–S1	100.803 ± 2.325	101.770	102.154
Sγ1–Fe1–S1	116.814 ± 4.363	118.783	119.24
Sγ1–Fe1–S2	109.507 ± 4.636	103.254	103.195
Sγ2–Fe1–S1	107.325 ± 4.634	110.876	110.851
Sγ2–Fe1–S2	111.045 ± 4.356	116.955	118.842
Sγ1–Fe1–Sγ2	110.402 ± 4.549	105.542	103.208
Sγ3–Fe2–S1	116.625 ± 3.839	112.659	114.314
Sγ3–Fe2–S2	107.502 ± 4.177	111.653	111.362
Sγ4–Fe2–S1	111.890 ± 3.953	118.650	114.901
Sγ4–Fe2–S2	113.772 ± 3.709	104.524	105.573
Sγ3–Fe2–Sγ4	100.792 ± 4.469	104.781	104.954

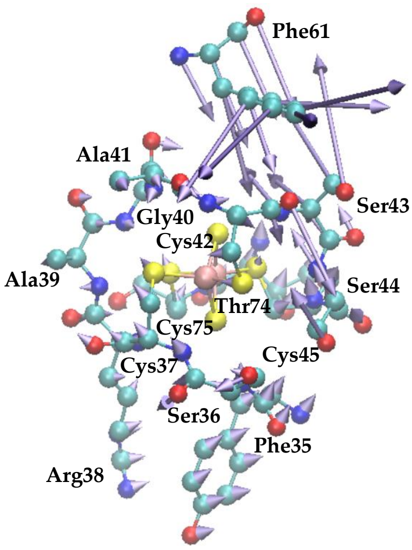

Figure S1. The dominant motion along PC1 up to 1.5 ns after the start of the production MD simulation.

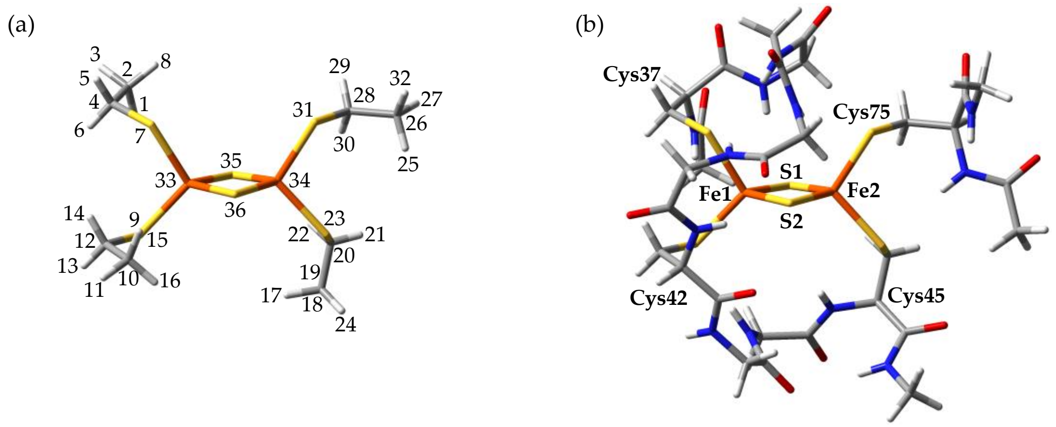

Figure S2. Geometry of Models 1 (a) and 2 (b) constructed based on the crystal structure by MCPB.py. The hydrogen, carbon, nitrogen, oxygen, sulfur, and iron atoms are displayed in white, gray, red, yellow, and orange, respectively, in tube models.

Table S3. Cartesian coordinates of the optimized geometry of Model 1 with oxidized state.

Number	Atomic symbol	*x*	*y*	*z*
1	H	−1.397372	−3.144321	1.853587
2	C	−2.452648	−3.414931	1.757874
3	H	−2.838263	−3.687151	2.750481
4	C	−3.234505	−2.251314	1.165126
5	H	−4.292635	−2.525356	1.067061
6	H	−3.185048	−1.385185	1.833102
7	S	−2.634861	−1.752167	−0.477720
8	H	−2.513620	−4.293598	1.107064
9	H	−1.885271	2.796419	−1.249317
10	C	−2.743176	3.131942	−0.660111
11	H	−3.523878	3.489570	−1.346178
12	C	−3.254227	1.989737	0.206021
13	H	−4.115576	2.326093	0.796974
14	H	−3.598676	1.163218	−0.424280
15	S	−2.000072	1.354598	1.359985
16	H	−2.413575	3.971505	−0.039012
17	H	1.062987	3.253768	−1.019125
18	C	1.612115	3.706257	−0.189257
19	H	0.940606	4.401827	0.330499
20	C	2.094956	2.615024	0.753217
21	H	2.632465	3.056992	1.601034
22	H	1.233752	2.079465	1.163798
23	S	3.209094	1.424553	−0.056215
24	H	2.456450	4.271034	−0.601259
25	H	4.113062	−1.433096	1.093438
26	C	4.529965	−2.332798	0.631719
27	H	4.821897	−3.032298	1.427495
28	C	3.507642	−2.965056	−0.300660
29	H	3.923646	−3.876125	−0.748823
30	H	2.618263	−3.252994	0.266996
31	S	2.995265	−1.879244	−1.666581
32	H	5.427069	−2.034523	0.079230
33	Fe	−0.947241	−0.263925	0.069620
34	Fe	1.740172	−0.272463	−0.598462
35	S	0.751056	−1.053851	1.237008
36	S	0.011008	0.483141	−1.770886

Table S4. Cartesian coordinates of the optimized geometry of Model 1 with reduced state.

Number	Atomic symbol	*x*	*y*	*z*
1	H	1.901285	−2.713471	−1.828501
2	C	2.797285	−3.247705	−1.497681
3	H	3.419824	−3.476670	−2.378011
4	C	3.549786	−2.396806	−0.482187
5	H	4.455148	−2.930545	−0.155525
6	H	3.867020	−1.468373	−0.970710
7	S	2.543498	−1.978531	0.973197
8	H	2.468103	−4.189740	−1.044342
9	H	2.823321	2.151715	1.644170
10	C	3.731305	2.402677	1.087222
11	H	4.569251	2.501077	1.796704
12	C	4.000561	1.326206	0.042559
13	H	4.914072	1.579439	−0.516563
14	H	4.178189	0.373094	0.554667
15	S	2.619233	1.112496	−1.120616
16	H	3.562238	3.373051	0.606400
17	H	−0.633650	3.518656	0.340257
18	C	−1.066870	3.963782	−0.559917
19	H	−0.263380	4.462814	−1.119934
20	C	−1.714254	2.865751	−1.391353
21	H	−2.129873	3.289804	−2.316458
22	H	−0.950931	2.137518	−1.683851
23	S	−3.041247	1.987372	−0.507816
24	H	−1.809556	4.713537	−0.257353
25	H	−4.252624	−0.802924	−1.304239
26	C	−4.823006	−1.604436	−0.825376
27	H	−5.100080	−2.347634	−1.589666
28	C	−3.985653	−2.233361	0.279559
29	H	−4.548264	−3.050698	0.753771
30	H	−3.077314	−2.658788	−0.157623
31	S	−3.491174	−1.055226	1.573524
32	H	−5.738651	−1.161673	−0.415831
33	Fe	1.046883	−0.251411	0.144744
34	Fe	−1.780538	0.193044	0.468229
35	S	−0.795422	−0.979108	−1.119803
36	S	−0.166561	0.927219	1.771642

Table S5. Input files for calculating oxidized *Cr*Fd1.

Atomic coordinates, atom types, and atomic charges for Fe1.

1	FE	-0.4320	-6.6080	34.1950	M1	1	FE1	0.955780

Atomic coordinates, atom types, and atomic charges for Fe2

1	FE	0.3480	-4.6240	35.9400	M2	1	FE2	0.943202

Atomic coordinates, atom types, and atomic charges for S1

1	S1	-0.0530	-4.4140	33.7700	Y3	1	S11	-0.768751

Atomic coordinates, atom types, and atomic charges for S2

1	S2	0.1360	-6.7640	36.3320	Y4	1	S21	-0.768751

Atomic coordinates, atom types, and atomic charges for Cys37

1	N	-2.4210	-5.4800	31.3650	N	1	CM1	-0.415700
2	H	-1.7280	-5.9320	31.6000	H	1	CM1	0.271900
3	CA	-3.6630	-5.9160	32.0090	CX	1	CM1	-0.335699
4	HA	-4.3370	-6.0010	31.3170	H1	1	CM1	0.184747
5	CB	-3.4910	-7.2800	32.6410	CT	1	CM1	-0.133559
6	HB2	-4.3720	-7.6680	32.7610	H1	1	CM1	0.158749
7	HB3	-3.0160	-7.8440	32.0110	H1	1	CM1	0.158749
8	SG	-2.6200	-7.3640	34.2360	Y1	1	CM1	-0.532185
9	C	-4.2140	-4.8740	32.9880	C	1	CM1	0.597300
10	O	-5.4230	-4.7640	33.1360	O	1	CM1	-0.567900

Atomic coordinates, atom types, and atomic charges for Cys42

1	N	-0.0600	-9.9780	35.4400	N	1	CM2	-0.415700
2	H	0.0290	-9.2800	35.9350	H	1	CM2	0.271900
3	CA	0.9670	-10.1510	34.4430	CX	1	CM2	0.005412
4	HA	1.1730	-11.0880	34.3020	H1	1	CM2	0.035745
5	CB	0.4580	-9.6580	33.0580	CT	1	CM2	-0.032968
6	HB2	0.9070	-10.1730	32.3700	H1	1	CM2	0.060571
7	HB3	-0.4880	-9.8620	32.9930	H1	1	CM2	0.060571
8	SG	0.6830	-7.9030	32.6880	Y2	1	CM2	-0.625555
9	C	2.2780	-9.5270	34.9430	C	1	CM2	0.597300
10	O	2.3440	-9.0920	36.0880	O	1	CM2	-0.567900

Atomic coordinates, atom types, and atomic charges for Cys45

1	N	4.0210	-4.3720	33.8900	N	1	CM3	-0.415700
2	H	3.4340	-4.8920	34.2440	H	1	CM3	0.271900
3	CA	4.2600	-3.1460	34.6480	CX	1	CM3	-0.470770
4	HA	4.6070	-2.4690	34.0460	H1	1	CM3	0.268333
5	CB	2.9230	-2.6920	35.2580	CT	1	CM3	0.037093
6	HB2	3.0140	-1.8050	35.6400	H1	1	CM3	0.101128
7	HB3	2.2430	-2.6350	34.5680	H1	1	CM3	0.101128
8	SG	2.4220	-3.8600	36.5240	Y5	1	CM3	-0.472064
9	C	5.3320	-3.3280	35.7450	C	1	CM3	0.597300
10	O	5.4710	-2.5060	36.6600	O	1	CM3	-0.567900

Atomic coordinates, atom types, and atomic charges for Cys75

1	N	1.0010	-1.4120	38.5340	N	1	CM4	-0.415700
2	H	1.3030	-2.1050	38.1240	H	1	CM4	0.271900
3	CA	-0.4310	-1.1000	38.2980	CX	1	CM4	-0.408052
4	HA	-0.4680	-0.1480	38.1170	H1	1	CM4	0.170050
5	CB	-0.9590	-1.8790	37.0750	CT	1	CM4	0.601561
6	HB2	-1.8230	-1.5240	36.8140	H1	1	CM4	-0.093969
7	HB3	-0.3590	-1.7470	36.3240	H1	1	CM4	-0.093969
8	SG	-1.1140	-3.6550	37.4210	Y6	1	CM4	-0.648927
9	C	-1.3290	-1.3110	39.5110	C	1	CM4	0.597300
10	O	-2.4810	-0.7950	39.4930	O	1	CM4	-0.567900

AMBER parameter file

```
MASS
M1 55.85                              Fe ion
M2 55.85                              Fe ion
Y1 32.06         2.900               S in cystine
Y2 32.06         2.900               S in cystine
Y5 32.06         2.900               S in cystine
Y6 32.06         2.900               S in cystine
Y3 32.06         2.900               Sp3 S connected with hydrogen
Y4 32.06         2.900               Sp3 S connected with hydrogen

BOND
M1-Y3  109.4    2.2070      Created by Seminario method using MCPB.py
M1-Y4  110.4    2.2054      Created by Seminario method using MCPB.py
M2-Y3   99.7    2.2266      Created by Seminario method using MCPB.py
M2-Y4  102.1    2.2216      Created by Seminario method using MCPB.py
Y1-M1   82.9    2.3157      Created by Seminario method using MCPB.py
Y2-M1   80.3    2.3223      Created by Seminario method using MCPB.py
Y5-M2   85.4    2.3090      Created by Seminario method using MCPB.py
Y6-M2   87.2    2.3017      Created by Seminario method using MCPB.py
CT-Y1  237.0    1.810       changed from 222.0 based on methanethiol nmodes
CT-Y2  237.0    1.810       changed from 222.0 based on methanethiol nmodes
CT-Y5  237.0    1.810       changed from 222.0 based on methanethiol nmodes
CT-Y6  237.0    1.810       changed from 222.0 based on methanethiol nmodes

ANGL
CT-Y1-M1    72.69     101.72    Created by Seminario method using MCPB.py
CT-Y2-M1    71.25     101.74    Created by Seminario method using MCPB.py
CT-Y5-M2    64.76     101.29    Created by Seminario method using MCPB.py
CT-Y6-M2    82.99     102.82    Created by Seminario method using MCPB.py
M1-Y3-M2    78.53      77.30    Created by Seminario method using MCPB.py
M1-Y4-M2    76.06      77.44    Created by Seminario method using MCPB.py
Y1-M1-Y2    30.15     104.41    Created by Seminario method using MCPB.py
Y1-M1-Y3    46.82     117.12    Created by Seminario method using MCPB.py
Y1-M1-Y4    52.03     109.70    Created by Seminario method using MCPB.py
Y2-M1-Y3    50.76     107.72    Created by Seminario method using MCPB.py
Y2-M1-Y4    44.75     115.13    Created by Seminario method using MCPB.py
Y4-M1-Y3    97.42     103.20    Created by Seminario method using MCPB.py
Y4-M2-Y3    99.30     102.04    Created by Seminario method using MCPB.py
Y5-M2-Y3    40.09     110.30    Created by Seminario method using MCPB.py
Y5-M2-Y4    43.36     111.66    Created by Seminario method using MCPB.py
Y5-M2-Y6    29.31     105.97    Created by Seminario method using MCPB.py
Y6-M2-Y3    53.17     112.32    Created by Seminario method using MCPB.py
Y6-M2-Y4    55.92     114.64    Created by Seminario method using MCPB.py
CX-CT-Y1    50.0      108.60    AA cys (was CT-CT-SH)
CX-CT-Y2    50.0      108.60    AA cys (was CT-CT-SH)
CX-CT-Y5    50.0      108.60    AA cys (was CT-CT-SH)
CX-CT-Y6    50.0      108.60    AA cys (was CT-CT-SH)
Y1-CT-H1    50.0      109.50    AA cyx     changed based on NMA nmodes
Y2-CT-H1    50.0      109.50    AA cyx     changed based on NMA nmodes
Y5-CT-H1    50.0      109.50    AA cyx     changed based on NMA nmodes
Y6-CT-H1    50.0      109.50    AA cyx     changed based on NMA nmodes

DIHE
CT-Y1-M1-Y2    3       0.00       0.00   3.0    Treat as zero by MCPB.py
CT-Y1-M1-Y3    3       0.00       0.00   3.0    Treat as zero by MCPB.py
CT-Y1-M1-Y4    3       0.00       0.00   3.0    Treat as zero by MCPB.py
CT-Y2-M1-Y3    3       0.00       0.00   3.0    Treat as zero by MCPB.py
CT-Y2-M1-Y4    3       0.00       0.00   3.0    Treat as zero by MCPB.py
CT-Y5-M2-Y3    3       0.00       0.00   3.0    Treat as zero by MCPB.py
CT-Y5-M2-Y4    3       0.00       0.00   3.0    Treat as zero by MCPB.py
CT-Y5-M2-Y6    3       0.00       0.00   3.0    Treat as zero by MCPB.py
CT-Y6-M2-Y3    3       0.00       0.00   3.0    Treat as zero by MCPB.py
CT-Y6-M2-Y4    3       0.00       0.00   3.0    Treat as zero by MCPB.py
M1-Y1-CT-H1    3       0.00       0.00   3.0    Treat as zero by MCPB.py
M1-Y2-CT-H1    3       0.00       0.00   3.0    Treat as zero by MCPB.py
M1-Y3-M2-Y4    3       0.00       0.00   3.0    Treat as zero by MCPB.py
M1-Y4-M2-Y3    3       0.00       0.00   3.0    Treat as zero by MCPB.py
M2-Y4-M1-Y3    3       0.00       0.00   3.0    Treat as zero by MCPB.py
M2-Y5-CT-H1    3       0.00       0.00   3.0    Treat as zero by MCPB.py
M2-Y6-CT-H1    3       0.00       0.00   3.0    Treat as zero by MCPB.py
CX-CT-Y1-M1    3       0.00       0.00   3.0    Treat as zero by MCPB.py
CX-CT-Y2-M1    3       0.00       0.00   3.0    Treat as zero by MCPB.py
CX-CT-Y5-M2    3       0.00       0.00   3.0    Treat as zero by MCPB.py
CX-CT-Y6-M2    3       0.00       0.00   3.0    Treat as zero by MCPB.py
Y1-M1-Y2-CT    3       0.00       0.00   3.0    Treat as zero by MCPB.py
Y1-M1-Y3-M2    3       0.00       0.00   3.0    Treat as zero by MCPB.py
```

```
Y1-M1-Y4-M2    3       0.00       0.00   3.0    Treat as zero by MCPB.py
Y2-M1-Y3-M2    3       0.00       0.00   3.0    Treat as zero by MCPB.py
Y2-M1-Y4-M2    3       0.00       0.00   3.0    Treat as zero by MCPB.py
Y4-M1-Y3-M2    3       0.00       0.00   3.0    Treat as zero by MCPB.py
Y5-M2-Y3-M1    3       0.00       0.00   3.0    Treat as zero by MCPB.py
Y5-M2-Y4-M1    3       0.00       0.00   3.0    Treat as zero by MCPB.py
Y5-M2-Y6-CT    3       0.00       0.00   3.0    Treat as zero by MCPB.py
Y6-M2-Y3-M1    3       0.00       0.00   3.0    Treat as zero by MCPB.py
Y6-M2-Y4-M1    3       0.00       0.00   3.0    Treat as zero by MCPB.py

IMPR

NONB
  M1          1.4000  0.0157074900       IOD set for Fe3+ ion OPC water from Li et al. JCTC, 2021, 17, 2342
  M2          1.4000  0.0157074900       IOD set for Fe3+ ion OPC water from Li et al. JCTC, 2021, 17, 2342
  Y1          1.9825  0.2824            gaff2
  Y2          1.9825  0.2824            gaff2
  Y5          1.9825  0.2824            gaff2
  Y6          1.9825  0.2824            gaff2
  Y3          2.0000  0.2500             W. Cornell CH3SH and CH3SCH3 FEP's
  Y4          2.0000  0.2500             W. Cornell CH3SH and CH3SCH3 FEP's
```

Table S6. Input files for calculating reduced *Cr*Fd1.

Atomic coordinates, atom types, and atomic charges for Fe1.

1	FE	-0.4320	-6.6080	34.1950	M1	1	FE1	1.289628

Atomic coordinates, atom types, and atomic charges for Fe2

1	FE	0.3480	-4.6240	35.9400	M2	1	FE2	0.878303

Atomic coordinates, atom types, and atomic charges for S1

1	S1	-0.0530	-4.4140	33.7700	Y3	1	S11	-1.020379

Atomic coordinates, atom types, and atomic charges for S2

1	S2	0.1360	-6.7640	36.3320	Y4	1	S21	-1.020379

Atomic coordinates, atom types, and atomic charges for Cys37

1	N	-2.4210	-5.4800	31.3650	N	1	CM1	-0.415700
2	H	-1.7280	-5.9320	31.6000	H	1	CM1	0.271900
3	CA	-3.6630	-5.9160	32.0090	CX	1	CM1	-0.583485
4	HA	-4.3370	-6.0010	31.3170	H1	1	CM1	0.224384
5	CB	-3.4910	-7.2800	32.6410	CT	1	CM1	0.068684
6	HB2	-4.3720	-7.6680	32.7610	H1	1	CM1	0.116657
7	HB3	-3.0160	-7.8440	32.0110	H1	1	CM1	0.116657
8	SG	-2.6200	-7.3640	34.2360	Y1	1	CM1	-0.772571
9	C	-4.2140	-4.8740	32.9880	C	1	CM1	0.597300
10	O	-5.4230	-4.7640	33.1360	O	1	CM1	-0.567900

Atomic coordinates, atom types, and atomic charges for Cys42

1	N	-0.0600	-9.9780	35.4400	N	1	CM2	-0.415700
2	H	0.0290	-9.2800	35.9350	H	1	CM2	0.271900
3	CA	0.9670	-10.1510	34.4430	CX	1	CM2	-0.077885
4	HA	1.1730	-11.0880	34.3020	H1	1	CM2	0.033021
5	CB	0.4580	-9.6580	33.0580	CT	1	CM2	0.036640
6	HB2	0.9070	-10.1730	32.3700	H1	1	CM2	0.014812
7	HB3	-0.4880	-9.8620	32.9930	H1	1	CM2	0.014812
8	SG	0.6830	-7.9030	32.6880	Y2	1	CM2	-0.828237
9	C	2.2780	-9.5270	34.9430	C	1	CM2	0.597300
10	O	2.3440	-9.0920	36.0880	O	1	CM2	-0.567900

Atomic coordinates, atom types, and atomic charges for Cys45

1	N	4.0210	-4.3720	33.8900	N	1	CM3	-0.415700
2	H	3.4340	-4.8920	34.2440	H	1	CM3	0.271900
3	CA	4.2600	-3.1460	34.6480	CX	1	CM3	-0.716522
4	HA	4.6070	-2.4690	34.0460	H1	1	CM3	0.333989
5	CB	2.9230	-2.6920	35.2580	CT	1	CM3	0.120888
6	HB2	3.0140	-1.8050	35.6400	H1	1	CM3	0.106035
7	HB3	2.2430	-2.6350	34.5680	H1	1	CM3	0.106035
8	SG	2.4220	-3.8600	36.5240	Y5	1	CM3	-0.430870
9	C	5.3320	-3.3280	35.7450	C	1	CM3	0.597300
10	O	5.4710	-2.5060	36.6600	O	1	CM3	-0.567900

Atomic coordinates, atom types, and atomic charges for Cys75

1	N	1.0010	-1.4120	38.5340	N	1	CM4	-0.415700
2	H	1.3030	-2.1050	38.1240	H	1	CM4	0.271900
3	CA	-0.4310	-1.1000	38.2980	CX	1	CM4	-0.658310
4	HA	-0.4680	-0.1480	38.1170	H1	1	CM4	0.236996
5	CB	-0.9590	-1.8790	37.0750	CT	1	CM4	0.817922
6	HB2	-1.8230	-1.5240	36.8140	H1	1	CM4	-0.144886
7	HB3	-0.3590	-1.7470	36.3240	H1	1	CM4	-0.144886
8	SG	-1.1140	-3.6550	37.4210	Y6	1	CM4	-0.659453
9	C	-1.3290	-1.3110	39.5110	C	1	CM4	0.597300
10	O	-2.4810	-0.7950	39.4930	O	1	CM4	-0.567900

AMBER parameter file

```
MASS
M1 55.85                              Fe ion
M2 55.85                              Fe ion
Y1 32.06         2.900               S in cystine
Y2 32.06         2.900               S in cystine
Y5 32.06         2.900               S in cystine
Y6 32.06         2.900               S in cystine
Y3 32.06         2.900               Sp3 S connected with hydrogen
Y4 32.06         2.900               Sp3 S connected with hydrogen

BOND
M1-Y3   59.6    2.3500      Created by Seminario method using MCPB.py
M1-Y4   60.0    2.3470      Created by Seminario method using MCPB.py
M2-Y3  111.5    2.2060      Created by Seminario method using MCPB.py
M2-Y4  115.2    2.2006      Created by Seminario method using MCPB.py
Y1-M1   40.5    2.4309      Created by Seminario method using MCPB.py
Y2-M1   38.3    2.4359      Created by Seminario method using MCPB.py
Y5-M2   56.3    2.4003      Created by Seminario method using MCPB.py
Y6-M2   58.8    2.3888      Created by Seminario method using MCPB.py
CT-Y1  237.0    1.810       changed from 222.0 based on methanethiol nmodes
CT-Y2  237.0    1.810       changed from 222.0 based on methanethiol nmodes
CT-Y5  237.0    1.810       changed from 222.0 based on methanethiol nmodes
CT-Y6  237.0    1.810       changed from 222.0 based on methanethiol nmodes

ANGL
CT-Y1-M1    79.57     103.38    Created by Seminario method using MCPB.py
CT-Y2-M1    80.82     102.94    Created by Seminario method using MCPB.py
CT-Y5-M2    62.88     100.09    Created by Seminario method using MCPB.py
CT-Y6-M2    88.28     101.77    Created by Seminario method using MCPB.py
M1-Y3-M2    58.70      78.36    Created by Seminario method using MCPB.py
M1-Y4-M2    57.18      78.53    Created by Seminario method using MCPB.py
Y1-M1-Y2    29.96     100.22    Created by Seminario method using MCPB.py
Y1-M1-Y3    47.66     116.49    Created by Seminario method using MCPB.py
Y1-M1-Y4    41.47     116.03    Created by Seminario method using MCPB.py
Y2-M1-Y3    39.40     113.57    Created by Seminario method using MCPB.py
Y2-M1-Y4    47.91     114.37    Created by Seminario method using MCPB.py
Y4-M1-Y3    94.89      97.08    Created by Seminario method using MCPB.py
Y4-M2-Y3    77.46     106.03    Created by Seminario method using MCPB.py
Y5-M2-Y3    34.18     109.82    Created by Seminario method using MCPB.py
Y5-M2-Y4    40.81     112.13    Created by Seminario method using MCPB.py
Y5-M2-Y6    32.25     101.69    Created by Seminario method using MCPB.py
Y6-M2-Y3    56.75     112.04    Created by Seminario method using MCPB.py
Y6-M2-Y4    56.04     115.18    Created by Seminario method using MCPB.py
CX-CT-Y1    50.0      108.60    AA cys (was CT-CT-SH)
CX-CT-Y2    50.0      108.60    AA cys (was CT-CT-SH)
CX-CT-Y5    50.0      108.60    AA cys (was CT-CT-SH)
CX-CT-Y6    50.0      108.60    AA cys (was CT-CT-SH)
Y1-CT-H1    50.0      109.50    AA cyx     changed based on NMA nmodes
Y2-CT-H1    50.0      109.50    AA cyx     changed based on NMA nmodes
Y5-CT-H1    50.0      109.50    AA cyx     changed based on NMA nmodes
Y6-CT-H1    50.0      109.50    AA cyx     changed based on NMA nmodes

DIHE
CT-Y1-M1-Y2    3       0.00       0.00   3.0    Treat as zero by MCPB.py
CT-Y1-M1-Y3    3       0.00       0.00   3.0    Treat as zero by MCPB.py
CT-Y1-M1-Y4    3       0.00       0.00   3.0    Treat as zero by MCPB.py
CT-Y2-M1-Y3    3       0.00       0.00   3.0    Treat as zero by MCPB.py
CT-Y2-M1-Y4    3       0.00       0.00   3.0    Treat as zero by MCPB.py
CT-Y5-M2-Y3    3       0.00       0.00   3.0    Treat as zero by MCPB.py
CT-Y5-M2-Y4    3       0.00       0.00   3.0    Treat as zero by MCPB.py
CT-Y5-M2-Y6    3       0.00       0.00   3.0    Treat as zero by MCPB.py
CT-Y6-M2-Y3    3       0.00       0.00   3.0    Treat as zero by MCPB.py
CT-Y6-M2-Y4    3       0.00       0.00   3.0    Treat as zero by MCPB.py
M1-Y1-CT-H1    3       0.00       0.00   3.0    Treat as zero by MCPB.py
M1-Y2-CT-H1    3       0.00       0.00   3.0    Treat as zero by MCPB.py
M1-Y3-M2-Y4    3       0.00       0.00   3.0    Treat as zero by MCPB.py
M1-Y4-M2-Y3    3       0.00       0.00   3.0    Treat as zero by MCPB.py
M2-Y4-M1-Y3    3       0.00       0.00   3.0    Treat as zero by MCPB.py
M2-Y5-CT-H1    3       0.00       0.00   3.0    Treat as zero by MCPB.py
M2-Y6-CT-H1    3       0.00       0.00   3.0    Treat as zero by MCPB.py
CX-CT-Y1-M1    3       0.00       0.00   3.0    Treat as zero by MCPB.py
CX-CT-Y2-M1    3       0.00       0.00   3.0    Treat as zero by MCPB.py
CX-CT-Y5-M2    3       0.00       0.00   3.0    Treat as zero by MCPB.py
CX-CT-Y6-M2    3       0.00       0.00   3.0    Treat as zero by MCPB.py
Y1-M1-Y2-CT    3       0.00       0.00   3.0    Treat as zero by MCPB.py
Y1-M1-Y3-M2    3       0.00       0.00   3.0    Treat as zero by MCPB.py
```

```
Y1-M1-Y4-M2    3       0.00       0.00   3.0    Treat as zero by MCPB.py
Y2-M1-Y3-M2    3       0.00       0.00   3.0    Treat as zero by MCPB.py
Y2-M1-Y4-M2    3       0.00       0.00   3.0    Treat as zero by MCPB.py
Y4-M1-Y3-M2    3       0.00       0.00   3.0    Treat as zero by MCPB.py
Y5-M2-Y3-M1    3       0.00       0.00   3.0    Treat as zero by MCPB.py
Y5-M2-Y4-M1    3       0.00       0.00   3.0    Treat as zero by MCPB.py
Y5-M2-Y6-CT    3       0.00       0.00   3.0    Treat as zero by MCPB.py
Y6-M2-Y3-M1    3       0.00       0.00   3.0    Treat as zero by MCPB.py
Y6-M2-Y4-M1    3       0.00       0.00   3.0    Treat as zero by MCPB.py

IMPR

NONB
  M1          1.4000  0.0157074900       IOD set for Fe3+ ion OPC water from Li et al. JCTC, 2021, 17, 2342
  M2          1.4000  0.0157074900       IOD set for Fe3+ ion OPC water from Li et al. JCTC, 2021, 17, 2342
  Y1          1.9825  0.2824            gaff2
  Y2          1.9825  0.2824            gaff2
  Y5          1.9825  0.2824            gaff2
  Y6          1.9825  0.2824            gaff2
  Y3          2.0000  0.2500             W. Cornell CH3SH and CH3SCH3 FEP's
  Y4          2.0000  0.2500             W. Cornell CH3SH and CH3SCH3 FEP's
```
